# Associations of Digital Clock Drawing Test with Subjective Cognitive Decline and Risk for Mild Cognitive Impairment and Dementia

**DOI:** 10.1002/alz70857_103366

**Published:** 2025-12-25

**Authors:** Moonil Kang, Cody Karjadi, Ting Fang Alvin Ang, Ashita S. Gurnani, Steven Lenio, Sherral A. Devine, Sanford H. Auerbach, Katherine A. Gifford, Kathryn L. Lunetta, Rhoda Au, Lindsay A. Farrer, Jesse Mez

**Affiliations:** ^1^ Framingham Heart Study, Boston University Chobanian & Avedisian School of Medicine, Boston, MA, USA; ^2^ Department of Medicine (Biomedical Genetics), Boston University Chobanian & Avedisian School of Medicine, Boston, MA, USA; ^3^ Department of Anatomy & Neurobiology, Boston University Chobanian & Avedisian School of Medicine, Boston, MA, USA; ^4^ Framingham Heart Study, Framingham, MA, USA; ^5^ Slone Epidemiology Center, Boston University Chobanian & Avedisian School of Medicine, Boston, MA, USA; ^6^ Boston University Chobanian & Avedisian School of Medicine, Boston, MA, USA; ^7^ Department of Neurology, Boston University Chobanian & Avedisian School of Medicine, Boston, MA, USA; ^8^ Boston University Alzheimer's Disease Research Center, Boston, MA, USA; ^9^ Department of Biostatistics, Boston University School of Public Health, Boston, MA, USA; ^10^ Biomedical Genetics, Department of Medicine, Boston University Medical School, Boston, MA, USA; ^11^ Department of Epidemiology, Boston University School of Public Health, Boston, MA, USA; ^12^ Alzheimer's Disease Research Center, Boston University Chobanian & Avedisian School of Medicine, Boston, MA, USA, Boston, MA, USA; ^13^ Department of Neurology and Ophthalmology, Boston University Chobanian & Avedisian School of Medicine, Boston, MA, USA

## Abstract

**Background:**

Digital cognitive assessment may offer earlier Alzheimer's disease (AD) and related dementias (ADRD) detection compared with traditional neuropsychological (NP) tests. Subjective cognitive decline (SCD) is recognized as a preclinical AD/ADRD feature. We hypothesized that among cognitively unimpaired (CU) individuals by traditional NP tests, the digital clock drawing test (dCDT) would be associated with concurrent SCD and with future objective cognitive impairment.

**Method:**

Participants from the Framingham Heart Study were followed longitudinally with assessment for SCD and NP testing, including dCDT, and were surveilled for mild cognitive impairment (MCI), AD, and all‐cause dementia. This study included CU participants at analytic baseline (first visit at/after age 60). Participants with SCD at baseline or in the future, defined by SCD‐plus criteria, were matched on age, sex, and education with participants without SCD. Using data from the latest CU visit, we conducted cross‐sectional analyses to test associations between dCDT (overall and four composite scores—drawing efficiency, information processing, simple and complex motor, spatial reasoning—in command and copy conditions) and SCD. Additionally, we examined associations between dCDT at baseline and time to MCI, AD, and all‐cause dementia. Models were adjusted for traditional NP performance (*i*.*e*., cognitive scores for executive function, language, memory), age, sex, education, *APOE4* and *APOE2* carrier status, and familial relatedness.

**Result:**

Among 1,601 participants (mean age at the latest CU visit: 71.6, women: 54.7%, SCD: 32.5%), 9.5%, 2.0%, and 2.6% developed MCI, AD, and all‐cause dementia, respectively. Better performance on dCDT—but not traditional NP tests—was associated with reduced odds of SCD: overall (OR=0.86, *p* = .007); drawing efficiency, command (OR=0.83, *p* = .002); information processing, command (OR=0.75, *p* = 1.14×10^‐5^) and copy (OR=0.83, *p* = .005). Better dCDT performance, after traditional NP performance adjustment, was associated with reduced hazards of future MCI and all‐cause dementia: overall dCDT (MCI: HR=0.88, *p* = .03; all‐cause dementia: HR=0.78, *p* = .02), drawing efficiency, command (MCI: HR=0.88, *p* = .03), and information processing, command (MCI: HR=0.85, *p* = .01; all‐cause dementia: HR=0.79, *p* = .03).

**Conclusion:**

In this community‐based study of CU individuals, dCDT was associated with SCD and time to MCI and all‐cause dementia, independent of traditional NP test performance. dCDT may capture subtle AD/ADRD cognitive changes not detected by traditional NP assessment.

